# An olfactory ‘stress test’ may detect preclinical Alzheimer’s disease

**DOI:** 10.1186/1471-2377-12-24

**Published:** 2012-05-02

**Authors:** Peter W Schofield, Houman Ebrahimi, Alison L Jones, Grant A Bateman, Sonya R Murray

**Affiliations:** 1 Neuropsychiatry service, Hunter New England Area Health, Newcastle, Australia & Centre for Translational Neuroscience and Mental Health, University of Newcastle, PO Box 833, Newcastle, NSW, 2300, Australia; 2 Department of Radiology, John Hunter Hospital, Newcastle, Australia; 3 Dean of Graduate Medicine, University of Wollongong, Newcastle, Australia; 4 Department of Radiology, John Hunter Hospital, Newcastle, Australia; 5 Neuropsychiatry service, Hunter New England Area Health, Newcastle, Australia

## Abstract

**Background:**

The olfactory bulb (OB) receives extensive cholinergic input from the basal forebrain and is affected very early in Alzheimer’s disease (AD). We speculated that an olfactory ‘stress test’ (OST), targeting the OB, might be used to unmask incipient AD. We investigated if change in olfactory performance following intranasal atropine was associated with several known antecedents or biomarkers of AD.

**Methods:**

We measured change in performance on the University of Pennsylvania Smell Identification Test (UPSIT) in the left nostril before (20-items) and after (remaining 20-items) intranasal administration of 1 mg of atropine. We administered cognitive tests, measured hippocampal volume from MRI scans and recorded Apolipoprotein E genotype as indices relevant to underlying AD.

**Results:**

In a convenience sample of 56 elderly individuals (14 probable AD, 13 cognitive impairment no dementia, 29 cognitively intact) the change in UPSIT score after atropine (‘atropine effect’ = AE) correlated significantly with demographically scaled episodic memory score (r = 0.57, p < 0.001) and left hippocampal volume (LHCV) (r = 0.53, p < 0.001). Among non-demented individuals (n = 42), AE correlated with episodic memory (r = 0.52, p < 0.001) and LHCV (r = 0.49, p < 0.001) and hierarchical linear regression models adjusted for age, gender, education, and baseline UPSIT showed that the AE explained more variance in memory performance (24%) than did LHCV (15%). The presence of any APOE ϵ4 allele was associated with a more negative AE (p = 0.014).

**Conclusions:**

The OST using atropine as an olfactory probe holds promise as a simple, inexpensive screen for early and preclinical AD and further work, including longitudinal studies, is needed to explore this possibility.

## Background

Recent clinico-pathological studies suggest that up to 40% of the non-demented elderly have Alzheimer’s disease(AD) pathology at autopsy in amounts sufficient to justify a neuropathological diagnosis of the disease [1]. It is plausible that such individuals would have progressed to clinically apparent AD had they lived long enough. A simple, inexpensive, widely-available test that could identify such individuals *in vivo* would facilitate the enrichment of investigational drug trials and would clearly have great value once disease-modifying agents for AD become available.

The neuropathological changes of AD arise initially within temporal lobe structures and the olfactory bulb (OB) [2,3]. Olfactory impairment is a characteristic feature of clinically-established AD which may arise earlier in its evolution [4-7]. Cholinergic neurons are especially vulnerable to the effects of β‐amyloid [8] which is produced in excess in AD and, at least in an animal model, has been shown to deposit in the OB very early in the disease [9]. The OB receives a large cholinergic input from the basal forebrain and modulation of cholinergic function at the level of the OB has been shown to significantly impact olfactory functioning [10,11]. In a recent study of individuals with AD, treatment with the cholinesterase inhibitor (i.e. pro-cholinergic) drug donepezil led to improvements in global functioning that were best predicted by treatment-related changes in *olfactory* test performance [12]. *Anticholinergics* cause exaggerated cognitive decline in those with AD relative to normal controls [13] and we hypothesized that such agents might also have an exaggerated impact on *olfactory* function in those with the disease. The OB is separated from the nasal cavity by the thin cribriform plate and a range of pharmacological agents have been shown to concentrate in the OB when administered intranasally [14,15]. We hypothesized that the anticholinergic drug atropine, given as a nasal spray, might concentrate in the OB where, by impacting cholinergic pathways already compromised, it could cause exaggerated reduction in olfactory performance in those with underlying AD pathology, and thus ‘unmask’ incipient AD. In which case, within an appropriate sample of elderly individuals, we would expect to find associations between the change in olfactory performance due to the atropine and other potential antecedents or biomarkers of AD, including Apolipoprotein E genotype, memory performance and hippocampal volume. Our study was designed to explore these associations.

## Methods

We recruited study participants through our memory disorders clinic, by referral from interested colleagues and word of mouth and they were seen over the period November 2009 to August 2010. Spouses and friends of patients were recruited as controls. Our intent was to assemble a sample of individuals spanning a spectrum from cognitively normal through to mildly demented due to AD. The study was approved by the Hunter New England Human Research Ethics Committee, and all participants provided written consent. Potential participants were all 65 years or older and lacked conditions other than AD likely to affect cognition, such as past traumatic brain injury, stroke, active psychiatric illness or major medical illnesses. Participants underwent clinical assessment comprising history, neurological examination and focused physical examination. The Mini Mental State Examination (MMSE)[16] and the Audio Recorded Cognitive Screen (ARCS), a cognitive assessment instrument that we have developed [17], were administered. The ARCS probes five cognitive domains (episodic memory, language, visuospatial function, fluency, and attention/executive function) and also generates an overall global score http://www.cognitionhealth.com. All raw scores can be scaled, based on normative data, according to age, gender and education, whereby expected (i.e. normal) performance is 100 (SD 15). The ARCS has good psychometric properties and has been used in a variety of clinical settings [17-19]. On the basis of the assessments above, participants were grouped into three categories: probable Alzheimer’s disease (AD) based on DSM IV [20] and NINCDS-ADRDA [21] criteria, cognitive impairment (CI), or normal control (NC). The diagnosis of CI was made when performance on any one of the cognitive domain scores on the ARCS fell more than 1.5 SD below appropriate norms for age, gender and education [17], and the individual did not meet criteria for dementia (general functioning was intact and MMSE was 24 or greater). Participants were adjudged normal if they did not meet criteria for either CI or dementia.

### The olfactory stress test (OST)

We used the well-validated 40-item University of Pennsylvania Smell Identification Test (UPSIT) [22] ‘scrcratch and sniff’ instrument in which odors are presented for recognition in a multiple-choice format. For the OST, 20 items of the UPSIT (UPSIT_20) were initially administered to the left nostril (with the right nostril occluded by a wad of cotton wool) after which 1 mg of atropine (0.1ml of 10mg/ml solution) was sprayed high into the left nostril. Atropine sulphate at 1 mg was chosen because its half-life is of a few hours, and because it is safely used at that dose intravenously in routine clinical practice. The patient then adopted a crouching head down position for one minute (the ‘Mecca Position’) to retain the spray. The remaining 20 items of the UPSIT were administered 40–45 min later through the left nostril, again with the right nostril occluded. The change in UPSIT score from baseline to post atropine, or ‘atropine effect’ (AE), represented an objective measure of the impact of atropine on olfactory functioning. Subjects were block randomised with respect to order of UPSIT (first or the second set of 20 items administered at baseline).The internal consistency correlation for 20-item fractions of the 40- item UPSIT is of the order of 0.86 [23].

### Manual hippocampal volumetry

Magnetic Resonance (MR) acquisitions were performed on a Siemens Avanto 1.5 T MR scanner (Siemens AG, Erlangen, Germany). A true inversion recovery sequence (TR 4000 ms, TE 373 ms, TI 350 ms) was used to provide strongly T1-weighted 2 mm coronal slices for manual measurement. Using the departmental PACS image viewer and with reference to a validated manual tracing method [24,25] the hippocampi were traced on each of the relevant images. Total hippocampal volume was obtained by summation of the area measurements of each tracing multiplied by the slice thickness. We chose to use left hippocampus for the purposes of analysis, this being the same side as the olfactory structures being challenged and bearing in mind that our ARCS instrument probes verbal episodic memory. Two independent raters (HE and CA) made measurements blinded to the clinical status of the study participant. For 10 randomly selected left hippocampi the inter-rater intraclass correlation was 0.78 and for 10 random remeasured left hippocampi the intra-rater intraclass correlations were r = 0.90 and 0.89 respectively.

### Correction for intracranial volume (ICV)

A 3-D T1-weighted gradient-echo sequence (MPRAGE, Siemens) was used with the FreeSurfer image analysis suite [26]. Up to eight simultaneous analyses were performed on an eight-core Mac Pro with 16 GB RAM (Apple Inc., Cupertino, CA) with an average *recon-all* time of 19 hours per subject http://surfer.nmr.mgh.harvard.edu.

Given the inherent difficulty of determining the CSF/skull interface on T1-weighted images, FreeSurfer uses an atlas normalization procedure to determine total intracranial volume. Although an estimate, this is a validated method for standardizing hippocampal volume for intracranial volume [27] which we did using the covariance approach described by Jack *et al.*[24].

Apolipoprotein E (APOE) genotyping for the presence of the three main alleles, ϵ4, ϵ3 and ϵ2 was conducted on all study participants.

### Statistics

SPSS version 19.0 was used for all analyses. Chi squared was used for comparison of categorical data. ANOVA was used to compare clinical groups with respect to basic demographic, cognitive and olfactory measures, with Scheffe followup pairwise comparisons. Univariate associations between AE, LHCV, baseline olfaction, APOE and cognitive measures were initially explored using Pearson correlations. Linear regression analyses were used to assess the associations between key independent variables (AE and LHCV) and memory, while adjusting for potential confounding variables. Logistic regression was used to quantify the association between APOE ϵ4 allele and dichotomous AE.

## Results

Table 1 summarises the characteristics of the sample by cognitive category. All participants were 65 years or older (mean 75.0, SD 6.0); mean years of education was 10.9 (SD 3.0); 25 (45%) of the subjects were males and 31 (55%) females. The initial sample comprised 29 cognitively normal (NC), 14 with cognitive impairment (CI) and 17 meeting clinical criteria for AD. Two individuals with dementia meeting clinical criteria for AD performed at chance level (5/20) on baseline testing with the UPSIT_20 and another was found to have lacunar infarcts bilaterally in the hippocampi (her atropine effect (AE) was 0). One participant with CI was noted to have changes of a right parietal lobe stroke that may have affected her cognition (her AE was also 0). These individuals were all excluded from further analyses. Three individuals declined MRI scanning, and two had contraindications to MRI scanning but all were retained in the sample, leaving 56 in all.

**Table 1:** Participant characteristics

	**NC*n*= 29**	**CI*n*= 13**	**AD*n*= 14**	**Significance*n*= 56**
**Age**	74.0 (6.6)	77.1 (5.6)	75.3 (4.6)	F_(2,53)_ = 1.3, *P* = 0.28
**Female n, (%)**	20 (69)	7 (54)	4 (29)	χ_(2)_^2^ =6.2, *P* = 0.04
**Education y (SD)**	10.6 (3.1)	11.3 (3.3)	11.1 (2.6)	F_(2,53)_ = 0.33, *P* = 0.77
**MMSE (SD)**	29.0 (1.6)	27.6 (2.4)	23.6 (4.0)	F_(2,53)_ = 21.0, *P* < 0.001, NC, CI > AD
**ARCSg**	107.2 (9.3)	84.7 (10.8)	57.2 (21.1)	F_(2,53)_ = 66.2,*P* < 0.001, NC > CI > AD
**Mem**	104.0 (11.5)	72.1 (19.9)	60.4 (12.9)	F_(2,53)_ = 52.7,*P* < 0.001, NC > CI, AD
**UPSIT_20**	14.3 (2.6)	14.6 (2.9)	10.4 (2.7)	F_(2,53)_ = 11.5, *P* < 0.001, NC, CI > AD
**AE**	0.28 (2.15)	−2.77 (2.71)	−2.43 (1.45)	F_(2,53)_ = 12.5, *P* < 0.001, NC > CI, AD
**LHCV**	1819 (370)	1410 (363)	1385 (293)	F_(2,48)_ = 9.1, *P* < 0.001, NC > CI, AD

Numbers shown represent mean (SD). NC: Normal Control; CI: Cognitive Impairment; AD: Alzheimer’s Disease; ARCSg: Scaled global score on the Audio Recorded Cognitive Screen (ARCS); Mem: Scaled memory domain score on the ARCS; UPSIT_20: Score on 20 items of the 40 item University of Pennsylvania Smell Identification Test; AE: Atropine Effect; LHCV: Left hippocampal volume adjusted for intracranial volume (cubic mm).

There were significant cognitive group-specific differences in MMSE (NC, CI > AD), global ARCS (NC > CI > AD), and scaled memory domain scores (NC > CI, AD). Mean baseline UPSIT scores (UP_20) did not differ between NC and CI, but both were significantly greater than that for the AD group (Table 1). By contrast, mean AE differed between NC and both CI and AD groups, but there were no group-specific differences in AE between CI and AD. Similar findings were present for left hippocampal volume, adjusted for ICV (LHCV), which was greater in the NC than in either CI or AD groups.

**Figure 1:**
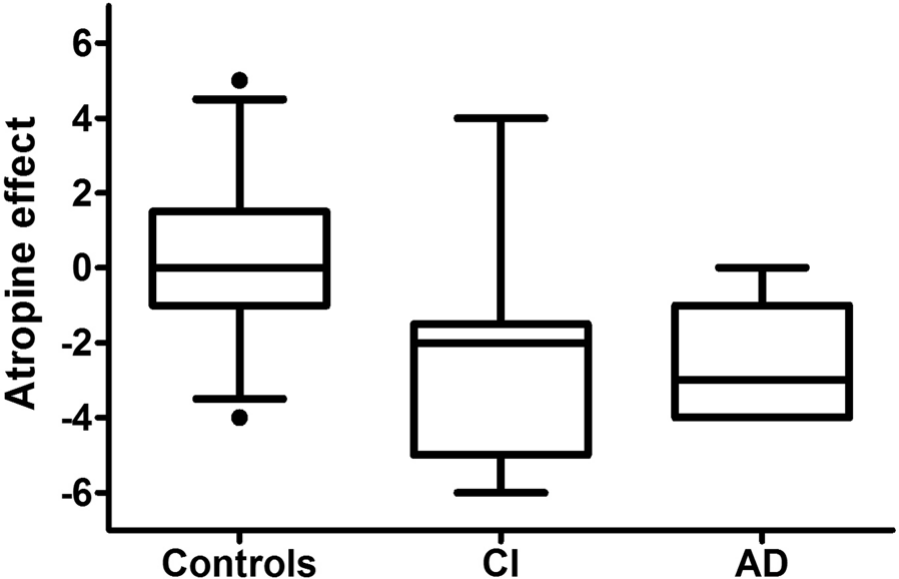
**Atropine effect by cognitive group.** NC: Normal Control; CI: Cognitive Impairment; AD: Alzheimer’s disease. Box & whiskers plots showing the median as a line and the boxes representing the inter-quartile range (25%-75%). Whiskers indicate 5-95 percentile. In terms of the atropine effect score, impaired study participants were much more like those with clinically diagnosed Alzheimer’s disease than controls, however there was broad overlap. AE < 0 was present in 9/29 (31%) NC, 12/13 (92%) with CI and 12/14 (86%) with AD. The rates of AE < 0 in the normal controls and individuals with AD in this study are very similar to the rates of underlying AD at autopsy reported in the literature in comparable clinical groups.

**Figure 2:**
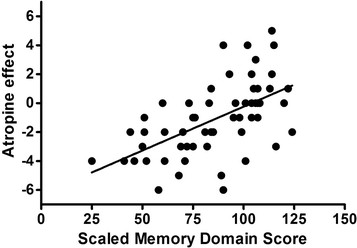
**Atropine effect vs. scaled memory domain score.** Almost without exception, low memory performance is associated with negative atropine effect (r = 0.57, *P <* 0.0001), but among those who perform well on memory performance there is a substantial range of atropine effect.

**Figure 3:**
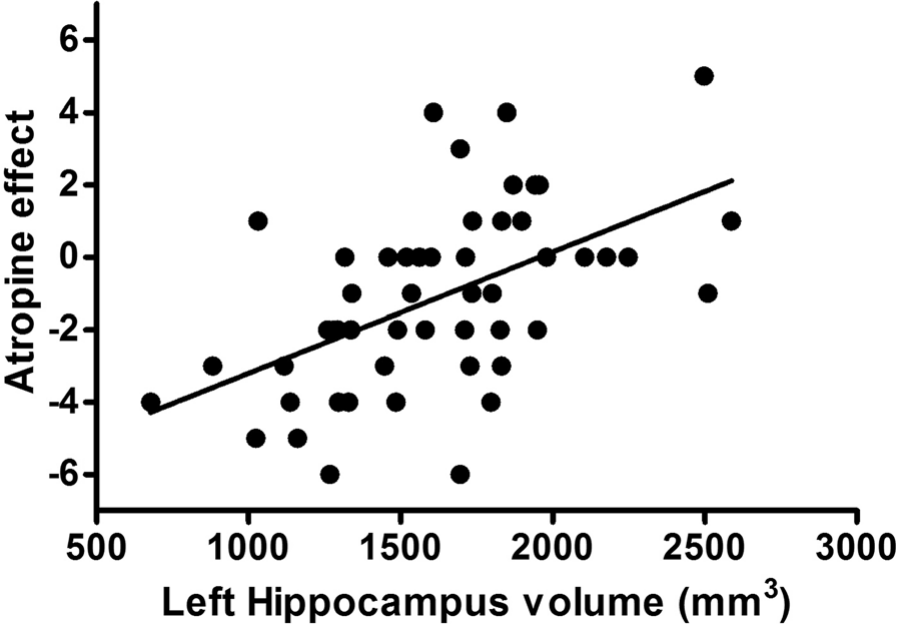
**Atropine effect vs. left hippocampal volume.** A strong relationship exists between atropine effect and hippocampal volume (r = 0.53, *P* = 0.0001) with more negative scores on AE associated with more atrophic hippocampi.

AE ranged from +5 to −6. Figure 1 depicts the distribution of AE by cognitive category. AE < 0 was present in 31% NC, 92% with CI and 86% with AD. Figure 2 shows the scatter plot of AE against scaled memory score. Figure 3 shows the scatter plot of AE and LHCV.

The univariate associations between AE, baseline UPSIT_20, LHCV, APOE genotype and scaled cognitive domain scores are depicted in Table 2. Correlations within the entire sample and in the non-demented sub-sample showed highly significant associations between AE, scaled memory domain score and LHCV. Baseline UPSIT_20 score was significantly associated with memory, but not with either LHCV or APOE genotype. Within the dementia sample, LHCV was significantly associated with overall cognition, memory, and visuospatial function.

**Table 2:** Pearson correlations between baseline UPSIT, AE, LHCV and APOE and cognitive test scores

	**All participants*n*= 56**									
	UP_20	APOE	LHCV	MMSE	ARCSg	Mem	Flu	Lang	VS	Atten
UPSIT_20	1	−0.04	0.17	0.43**	0.41**	0.39**	0.15	0.19	0.46**	0.40**
AE	0.06	−0.33*	0.53**	0.37**	0.51**	0.57**	0.36**	0.38**	0.28*	0.31*
LHCV	0.17	−0.12	1	0.45**	0.56**	0.57**	0.34*	0.40**	0.43**	0.28*
	**Non – Demented*n*= 42**									
UPSIT_20	1	−0.05	0.12	0.33*	0.12	0.11	−0.10	−0.02	0.25	0.17
AE	−0.15	−0.30	0.49**	0.22	0.50**	0.52**	0.31*	0.29	−0.02	0.21
LHCV	0.12	−0.08	1	0.29	0.44**	0.47**	0.14	0.23	0.23	0.15
	**AD*n*= 14**									
UPSIT_20	1	0.28	−0.53	−0.17	−0.31	−0.04	−0.42	−0.34	−0.07	0.18
AE	−0.05	−0.41	0.52	0.50	0.48	0.47	0.16	0.38	0.46	−0.03
LHCV	−0.53	−0.16	1	0.58*	0.80**	0.71**	0.48	0.54	0.59*	0.10

* *P* < 0.05; ** *P* < 0.01; UP_20: Score on 20 items of the 40 item University of Pennsylvania Smell Identification Test (UPSIT_20); AE: Atropine Effect; LHCV: Left hippocampal volume adjusted for intracranial volume; APOE: Apolipoprotein E allele dichotomised (no ϵ4 = 0, any ϵ4 = 1); MMSE: Mini Mental State Examination; ARCSg: Scaled global score on the Audio Recorded Cognitive Screen (ARCS); Mem: Scaled memory domain score on the ARCS; Flu: Scaled fluency domain score on the ARCS; Lang: Scaled language domain score on the ARCS; VS: Scaled visuospatial domain score on the ARCS; Atten: Scaled attention/executive domain score on the ARCS.

We conducted a series of linear regression analyses within the non-demented sample with memory domain score as the outcome. (See Table 3). In hierarchical regression analyses, age, gender and education were entered together, then baseline UPSIT_20 score and then either LHCV (Model 1) or AE (Model 2) separately. AE explained more variance (24%) in memory scores than did LHCV (15%). Next, we entered the same 4 initial variables, then AE followed by LHCV (Model 3). In this model, LHCV explained only 2% additional variance in memory, after accounting for that explained by AE. By contrast, in Model 4 in which AE was entered *after* LHCV, AE accounted for an additional 15% in the variance: equal to that explained by LHCV in the same model. Analytic models run without baseline UPSIT_20 score gave essentially the same results. Finally, in a linear regression model predicting memory domain score with age, gender, education, LHCV and AE entered simultaneously, AE (t = 3.3, p = 0.003) but not LHCV (t = 1.2, p = 0.24) retained significance.

**Table 3:** Hierarchical linear regression models predicting memory domain score

**Independent variable**	**ΔR**^**2**^	**ΔF**	**Standardised Beta**	***P*value**
**Model 1 (R**^**2**^**=0.39)**				
LHCV	0.15	7.9	0.42	0.008
**Model 2 (R**^**2**^**= 0.49)**				
AE	0.24	17.1	0.51	0.0002
**Model 3 (R**^**2**^**= 0.54)**				
AE	0.28	19.2	0.54	0.0001
LHCV	0.02	1.28	0.17	0.27
**Model 4 (R**^**2**^**= 0.54)**				
LHCV	0.147	7.9	0.42	0.008
AE	0.151	10.5	0.46	0.003

LHCV: Left hippocampal volume adjusted for intracranial volume; AE: Atropine Effect.

In all models, age, gender, education were entered first, then baseline UPSIT. In model 3, AE was entered next, followed by LHCV. In model 4, this order was reversed. Restricted to non-demented (*n* = 39 for analyses including LHCV, *n* = 42 for model 2).

### Apolipoprotein E associations

One or more APOE ϵ4 alleles were present in 50% of individuals with dementia, 46% with CI, and 31% NC (χ^2^ =1.76, p = 0.41). Relative to no ϵ4, the presence of ϵ4 was associated with lower mean AE in the entire sample (any ϵ4: -2.14, no ϵ4: -0.44, *t*-test, p = 0.014), in non-demented individuals (any ϵ4: -1.73, no ϵ4: -0.07, p = 0.056) and in those with dementia (any ϵ4: -3.00, no ϵ4: -1.86, p = 0.15). The percentages of AE <0 in the NC (31%) and AD (86%) subgroups were very similar to the respective rates of AD pathology at autopsy in cognitively normal samples and clinically diagnosed AD, so we chose AE <0 as potentially indicative of underlying AD pathology for the purposes of exploratory analyses. Within the entire sample, with the AE < 0 as the outcome in a binary logistic regression adjusted for age, any APOE ϵ4 (relative to no ϵ4) was associated with significantly increased risk (Odds Ratio (OR) 3.53, 95% confidence interval 1.09-11.38). Limiting the analysis to the non-demented sample (OR 3.17, 95% confidence interval 0.84-11.94) slightly reduced the odds, with loss of statistical significance.

Finally, in analyses stratified by APOE status, correlations between AE and scaled memory score (no ϵ4 (n = 34) r = 0.58, p < 0.001; any ϵ4 (n = 22) r = 0.49, p < 0.05), and AE and LHCV (no ϵ4 (n = 30) r = 0.56, p = 0.001; any ϵ4 (n = 21) r = 0.44, p < 0.05), retained statistical significance.

## Discussion

In this study, the change in performance on a standard olfactory identification test, following an intranasal anticholinergic challenge, (i.e. the AE), correlated strongly with several well-recognized biomarkers or antecedents of AD. These associations were significantly stronger than those between baseline (i.e. conventional) olfactory testing and the relevant biomarkers, and were preserved when the analytic sample was restricted to non-demented study participants. Linear regression analyses showed that the AE explained more variance in memory performance in non-demented individuals than did hippocampal volume, inviting speculation that the AE might represent a proxy for a process more salient than hippocampal atrophy in the early stages of AD.

In previous, larger studies of elderly individuals the results of conventional olfactory testing (i.e. comparable to our baseline testing during the OST) have been shown to correlate only modestly with cognitive performance and hippocampal volume. Among 1092 non-demented elderly participants in a recent community-based study [7], UPSIT scores correlated with delayed recall (r = 0.28) and, in a subsample of 571, hippocampal volume (r = 0.16), comparable to the estimates we obtained between baseline UPSIT and memory (r = 0.11) and hippocampal volume (r = 0.12) among non-demented individuals in the current study but substantially less than the correlations between AE and memory (r = 0.52) and hippocampal volume (r = 0.49) within the same sample. Other studies have examined the value of conventional olfactory testing for predicting subsequent cognitive decline [5,6,28,29]. Of these, several have demonstrated an interaction between olfaction and APOE genotype status indicating that the predictive value of olfactory testing may be restricted largely to individuals who are APOE ϵ4 positive [6,28]. Presumably, this reflects the greater probability that any olfactory decline is due to underlying AD (rather than to other non-specific local nasal pathology) in those at increased genetic risk for this condition, relative to the situation in APOE ϵ4 negative individuals. By contrast, correlations between AE of the OST and cognitive measures and hippocampal volume in the current study were at least as strong within the APOE ϵ4-negative sample relative to ϵ4-positive individuals. Together, the above results suggest that the OST (AE) may be more sensitive and specific for underlying AD pathology than is conventional olfactory testing.

Clinicopathologic studies of non-demented individuals have shown associations between antemortem cognition, particularly episodic memory, and the ‘burden’ of AD pathology at autopsy [1,30]. In one study, the strongest association was between limbic ‘diffuse senile plaques’ and logical memory (r = −0.58) [1]. Mortimer *et al*. [30] found associations between neurofibrillary counts and Braak stage (as indices of AD pathology) [2] and delayed memory one year prior to death. When hippocampal volume was added to the linear regression models, it alone remained a significant predictor of memory, suggesting that the effect of the neuropathology was mediated through hippocampal atrophy [30]. Similar conclusions have been reached from studies using Pittsburgh Compound B (PiB) Positron Emission Tomography (PET) imaging to detect insoluble amyloid deposits in the brain. In one such study, the PiB index of amyloid deposition within a combined sample of normal controls and PiB + MCI subjects was significantly associated with both episodic memory and hippocampal volume (HCV) [31]. However, when PiB index and HCV were both entered into a regression model predicting episodic memory, only HCV was significant. Our findings stand in direct contrast to these results. AE but not LHCV remained as the significant predictor of episodic memory in analyses, similar to those described above, in which both variables were included. This suggests that AE is a proxy for a process that subsumes hippocampal atrophy in the evolution of AD. Biological plausibility for the relevance of AE with respect to AD pathology is further supported by the strength of the relationship between AE < 0 and the APOE genotype. The odds ratios we obtained were very similar to the increased risk of AD due to APOE ϵ4 that has been estimated from clinical and pathological studies [32,33].

Structural and functional mechanisms warrant consideration in relation to the findings we report. The OB receives massive cholinergic input from the basal forebrain [34]. Neuropathological studies have consistently demonstrated a profound loss of cholinergic neurons in the Nucleus of Meynert (Ch4) in the presence of other pathological features of AD [35,36]. Similar changes in the adjacent Ch3 nucleus, which provides the rich cholinergic innervation to OB, have also been documented [10,37]. Damage to cholinergic structures occurs early in the evolution of AD, although how early, and to what extent, remains contentious [35]. Physiologically relevant concentrations of β-amyloid specifically interfere with cholinergic neurons and neurotransmission [36]. In neuronal culture/in vitro studies, β-amyloid has been shown to reduce high affinity choline uptake, decrease the rate of acetylcholine (ACh) synthesis, inhibit ACh release, and impair muscarinic receptor activation of G proteins [8,36]. Elegant studies by Bales and colleagues produced very direct and compelling evidence for the negative impact of β-amyloid on cholinergic function [38]. These workers measured ACh release within the hippocampus using an in vivo microdialysis technique in awake, moving mice. Measurements were made in PDAPP transgenic (a well-characterized model of AD in which β-amyloid is over-expressed) and wild type (WT) mice. Relative to WT, PDAPP mice had significantly lower basal production of ACh. When the experimental animals were injected with the pan-muscarinic receptor antagonist scopolamine, WT mice showed a seven-fold increase over basal rate in hippocampal ACh production, but the response in transgenic animals was very significantly blunted. Finally, when PDAPP mice were pre-treated with a monoclonal anti β‐amyloid antibody (m266), the deficient, blunted ACh response to scopolamine was normalised [38]. The results implicate soluble β-amyloid in the differential response of cholinergic neurons to an anticholinergic. *Soluble* β-amyloid within olfactory structures, perhaps the OB specifically, might account via similar mechanisms for the results we have obtained in the current study. Specifically, post-synaptic cholinergic blockade might be overcome by enhanced ACh release in normals but the absence of such a response, related to amyloid, could explain a reduction in olfactory performance from variable degrees of transient cholinergic transmission failure. Noteworthy in this context, Wesson et al. have recently shown in the Tg2576 (APP over-expressing) mouse model that soluble amyloid appears earlier in the OB than in any other brain region, and is associated with olfactory deficits [9]. The soluble form of β-amyloid, including oligomers of dimers and trimers, rather than the insoluble fibrillary form, such as is detected by PiB imaging, may be most toxic [39,40], is present before the development of amyloid plaques [41], and has been shown to correlate better with cognition than does fibrillary amyloid [42].

There are both uncertainties and important limitations in relation to our study and it would be premature and inappropriate at this point to apply the OST in a clinical context to aid in diagnosis or prognostication. Based on its modest molecular weight, lipophilic properties, and the fact that it is known to cross the blood brain barrier, we hypothesized that atropine sulphate would concentrate in the OB when delivered intranasally, as other small drugs have been shown to do [15]. However, we have no *direct* evidence either from the literature or from our own study to support that. It is possible, for example, that the atropine effect we have observed reflects the consequences of *systemically*-absorbed atropine operating more diffusely on cholinergic pathways within the olfactory sytem. We did not administer a control, pharmacologically inactive, nasal spray to any of our participants and it could be questioned whether our results could have arisen due to some non-specific effect of a nasal instillation. In the absence of data, we cannot absolutely refute such a criticism. However, based on the coherence of the findings, all predicted a priori, of associations between atropine effect and each of memory, hippocampal volume and APOE genotype, it seems implausible that these could be accounted for by some chance effect not under pinned by relevant biology. In future studies, we will additionally administer a non-active spray to a subset of study participants to address this concern directly. The current sample was relatively small. Inter- and intra-rater reliability of hippocampal volume estimation was lower than has sometimes been reported in the literature [7] and it is possible that measurement error may have diminished the apparent association between hippocampal volume and other measures. In particular, this may have inflated the contribution of AE, relative to hippocampal volume, in our regression analyses predicting memory score. However, the correlations we obtained between hippocampal volume and baseline UPSIT score, and between hippocampal volume and memory score, were very similar to those obtained in other mixed samples of cognitively normal and impaired individuals [7,43]. The current study does not speak to the specificity of the OST in terms of aetiology because we deliberately excluded individuals in whom conditions other than AD might have contributed to cognitive decline. We are undertaking further studies with larger samples, more diagnostic heterogeneity and longitudinal followup to better characterize the properties of the OST. The results of such studies would need to be carefully evaluated before consideration could be given to the use of the OST in clinical practice.

Finally, we note that the OB is a complex structure which contributes to olfactory processing by engaging at least 20 different neurotransmitters [4] and it seems plausible that the olfactory stress test technique could serve more broadly as a ‘window on the brain’. Appropriate pharmacological probes could potentially be chosen for intranasal administration to target specific neurotransmitters known to be implicated both in olfactory functioning and a neuropsychiatric condition of interest. For example, we are currently examining the effects of intranasal methylphenidate and ketamine on olfactory functioning in controls and individuals with schizophrenia seeking group-specific differences that might be salient.

## Conclusion

A simple, inexpensive ‘stress test’ of olfaction warrants further evaluation as a possible screen for early and preclinical Alzheimer’s disease. More generally, the approach we have outlined potentially could, using appropriate pharmacological stressors, constitute a window on the brain for early detection of, or identification of vulnerability for, other neuropsychiatric conditions in which olfactory disturbances are characteristic.

## Competing interests

Dr Schofield is the inventor of the Olfactory Stress Test for which a provisional patent has been lodged. Dr Schofield is also an inventor of the Audio Recorded Cognitive Screen used in this study. The remaining authors report no financial interests or potential conflicts of interest.

## Authors’ contributions

PS conceived the olfactory stress test, and was responsible for study design, clinical assessment of participants, analyses, and writing the manuscript. HE established the Freesurfer capability for this study and was responsible for manual hippocampal measurements and critical review of the manuscript. GB contributed to study design and critically reviewed the manuscript. AJ contributed to study design and critically reviewed the manuscript. SM was responsible for administering the olfactory stress test, data entry, and critical review of the manuscript.

## Pre-publication history

The pre-publication history for this paper can be accessed here:

http://www.biomedcentral.com/1471-2377/12/24/prepub
